# Sub-optimal presentation of painful facial expressions enhances readiness for action and pain perception following electrocutaneous stimulation

**DOI:** 10.3389/fpsyg.2015.00913

**Published:** 2015-07-07

**Authors:** Ali Khatibi, Martien Schrooten, Katrien Bosmans, Stephanie Volders, Johan W.S. Vlaeyen, Eva Van den Bussche

**Affiliations:** ^1^Research Group on Health Psychology, Katholieke Universiteit LeuvenLeuven, Belgium; ^2^Laboratory of Research on Neuropsychology of Pain, University of MontrealMontréal, QC, Canada; ^3^Center for Health and Medical Psychology, Örebro UniversityÖrebro, Sweden; ^4^Department of Psychology, Vrije Universiteit BrusselBrussels, Belgium; ^5^Department of Clinical Psychological Science, Maastricht UniversityMaastricht, Netherlands

**Keywords:** painful facial expressions, observation of pain, sub-optimal processing, action readiness, pain perception

## Abstract

Observation of others’ painful facial expressions has been shown to facilitate behavioral response tendencies and to increase pain perception in the observer. However, in previous studies, expressions were clearly visible to the observer and none of those studies investigated the effect of presence of peripheral stimulation on response tendencies. This study focuses on the effect of sub-optimal presentation of painful facial expressions in the presence and absence of an electrocutaneous stimulus. Twenty-two healthy individuals categorized arrow targets which were preceded by a sub-optimally presented facial expression (painful, happy, or neutral in different blocks). On half of the trials, aversive electrocutaneous stimulation was delivered to the wrist of the non-dominant hand between the presentation of facial expression and target (an arrow directing to right or left). Participants’ task was to indicate direction of the arrow as soon as it appears on the screen by pressing the corresponding key on the keyboard and to rate their pain at the end of block. Analysis showed that responses were faster to targets preceded by aversive stimulation than to targets not preceded by stimulation, especially following painful expressions. Painfulness ratings were higher following painful expressions than following happy expressions. These findings suggest that sub-optimally presented painful expressions can enhance readiness to act to neutral, non-pain-related targets after aversive stimulation and can increase pain perception.

## Introduction

Facial expressions of pain are salient social signals of potential physical threat (Williams, 2002). It has been recently re-emphasized that the consequences of pain expressions could potentially be profound, not only for the sufferer, but also for the observer (Hadjistavropoulos et al., 2011). For example, observation of pain in others may elicit empathy and fear responses in the observer, associated with hypervigilance to threat, increased urge for avoidance of pain/threat-related signals, and elevated perception of pain in the observer (Goubert et al., 2005; Khatibi et al., 2014).

Indeed, there are some indications that the observation of others’ painful facial expressions has an effect on responses to pain among healthy individuals. The observation of pain in the faces of other people increases the observer’s nociceptive flexion reflex (NFR) in response to a painful electrocutaneous stimulus, which has been taken to reflect an elevated readiness for taking (avoidance) action (Vachon-Presseau et al., 2011; Mailhot et al., 2012; Khatibi et al., 2014). In addition, the observation of others’ painful facial expressions has been shown to have an effect on pain perception in healthy individuals. More specifically, observing painful facial expressions increased perceived unpleasantness of an electrocutaneous stimulus but had no effect on perceived intensity (Vachon-Presseau et al., 2011; Mailhot et al., 2012; Khatibi et al., 2014). Observing painful facial expressions, as compared to observing neutral, joyful, or fearful expressions, also increased perception of thermally induced pain (Reicherts et al., 2013).

In all aforementioned studies on the impact of the observation of painful facial expressions on readiness for action or pain perception so far, expressions were presented in optimal visual conditions, and were therefore clearly visible to the observer. They draw the attention to the capacity for the understanding of the affective state of others and its contribution to the preparation of appropriate reaction (Jackson et al., 2005). On the other hand, there are studies suggesting that conscious processing of emotions is not necessary and the neural system’s response to the emotional expression of others does not rely on the explicit processing of expressions and is reflective in nature (Davis and Whalen, 2001). Considering pain as an emotional experience, it is unknown whether conscious processing of facial expressions is necessary for the facilitation of responses, or whether semantic, non-social processing of emotion in the expression alone can influence readiness for action. In the present study, we aimed to investigate the effect of *sub-optimal* presentation of painful facial expressions on readiness for action in healthy individuals. Previous studies have shown that sub-optimally presented stimuli can be processed semantically and can influence our behavior (Van den Bussche et al., 2009; Schrooten et al., 2011). So it can be expected that also sub-optimally presented painful facial expressions could prime behavioral responses.

Few previous studies investigated the interaction between stimuli from two modalities and its effect on the preparation of actions. For example, Mulckhuyse and Crombez (2014) have shown that congruent presentation of spatial cues (visual) and peripheral cues (electrocutaneous stimulation) can result in stronger action preparation and faster responses to a target. They suggested that electrical stimulation decreased reaction time (RT) by improving action preparation and stronger congruency effect is due to the response priming effect. However, they did not take the effect of emotional factors into account. In the current study, we were interested to see whether sub-optimally presented painful expressions that are followed by painful electrocutaneous stimulation can increase readiness for taking an action in comparison with the situation in which there is no electrocutaneous stimulation. We expect that participants show an increased readiness for action (indicated by faster responses on a non-pain-related task) on trials with electrocutaneous stimulation as compared to trials without stimulation, and that this facilitation is stronger after sub-optimally presented painful expressions, as compared to sub-optimally presented happy or neutral expressions. Furthermore, along with findings of previous studies, which suggested that processing of pain in facial expression of other people under optimal condition improves the observer’s perceived pain (Vachon-Presseau et al., 2011; Mailhot et al., 2012; Khatibi et al., 2014), we hypothesize that processing of painful facial expressions under sub-optimal condition will lead to increased pain ratings of an electrocutaneous stimulus compared to the happy or neutral expressions.

## Materials and Methods

### Participants

Twenty-two healthy volunteers (six males), with a mean age of 25.6 years (SD = 3.8, range 22–35) participated in the study. Exclusion criteria were current pain complaints, pregnancy, and electronic implants. All participants had Dutch as mother tongue. All had normal (or corrected to normal) vision. The study was approved by the Medical Ethics Committee of the Vrije Universiteit Brussel (reference 2011/197).

### Questionnaires

#### Pain Catastrophizing Scale

The Pain Catastrophizing Scale (PCS; Dutch version: Van Damme et al., 2000, 2002a,b) consists of 13 items which describe different thoughts and feelings that may be associated with pain. Participants indicate the degree to which they experience each of those thoughts and feelings when they feel pain on a 5-point Likert scale (0 = not at all; 4 = all the time). Higher PCS total scores reflect higher levels of trait catastrophizing about pain. The PCS has three subscales with items referring to thoughts or feelings associated with magnification, rumination, or helplessness. The PCS has demonstrated good psychometric properties, also for healthy Dutch speaking populations (Van Damme et al., 2000, 2002a).

#### Fear of Pain Questionnaire

The Fear of Pain Questionnaire [FPQ-III; (McNeil and Rainwater, 1998) Dutch version: (Roelofs et al., 2005)] consists of 30 items that describe pain-arousing experiences. Participants indicate their fear for those experiences on a 5-point Likert scale (1 = not at all; 5 = extreme). Higher FPQ-III total scores reflect higher levels of trait fear of pain. The FPQ-III has three subscales with items referring to experiences of severe pain, minor pain, or medical pain. The FPQ-III has demonstrated good psychometric properties, also for healthy Dutch speaking populations (Roelofs et al., 2005).

### Task Material

#### Electrocutaneous Stimuli

The electrocutaneous stimulus (2-ms duration, rectangular waveform, Frequency = 65 Hz) was delivered by a constant current stimulator (DS7A, Digitimer, Welwyn Garden City, England) using surface sensormedics electrodes (8 mm) filled with K–Y gel attached to the back of *non*-*dominant* hand (Meulders and Vlaeyen, 2013). Stimulus intensity was individually set using a work-up procedure (Meulders and Vlaeyen, 2013). A series of stepwise increasing intensities of electrocutaneous stimuli (2 mA increase per step) was delivered once. Participants were asked to rate the painfulness of each stimulus upon stimulus delivery on an 11-point Likert scale (0 = “not painful at all”, 10 = “Extremely painful”). Intensities were increased to a level that was reported as painful but just tolerable as reported by the participant. The highest intensity presented during this procedure was used during the priming task. Mean painfulness rating of the selected stimuli was 6.7 (SD = 0.8; range: 6–8).

#### Facial Expressions

Grayscale photographs (width 6 cm, height 4.5 cm) of three types of facial expressions were used: four painful expressions, four happy expressions, and four neutral expressions. The expressions were from four different actors (two females, two males) with the three types of expressions for each actor. The expressions were snapshots of dynamic facial expressions (1-sec movies) and were selected from an existing database (Simon et al., 2008). Selection of expressions was based on intensity ratings acquired from authors of a previously published study (Vachon-Presseau et al., 2011). On all photographs, head and eye-gaze were directed forward and the head filled most of the picture. See supplementary material for the photographs included in the current study.

### Tasks

#### Priming Task

**FIGURE 1:**
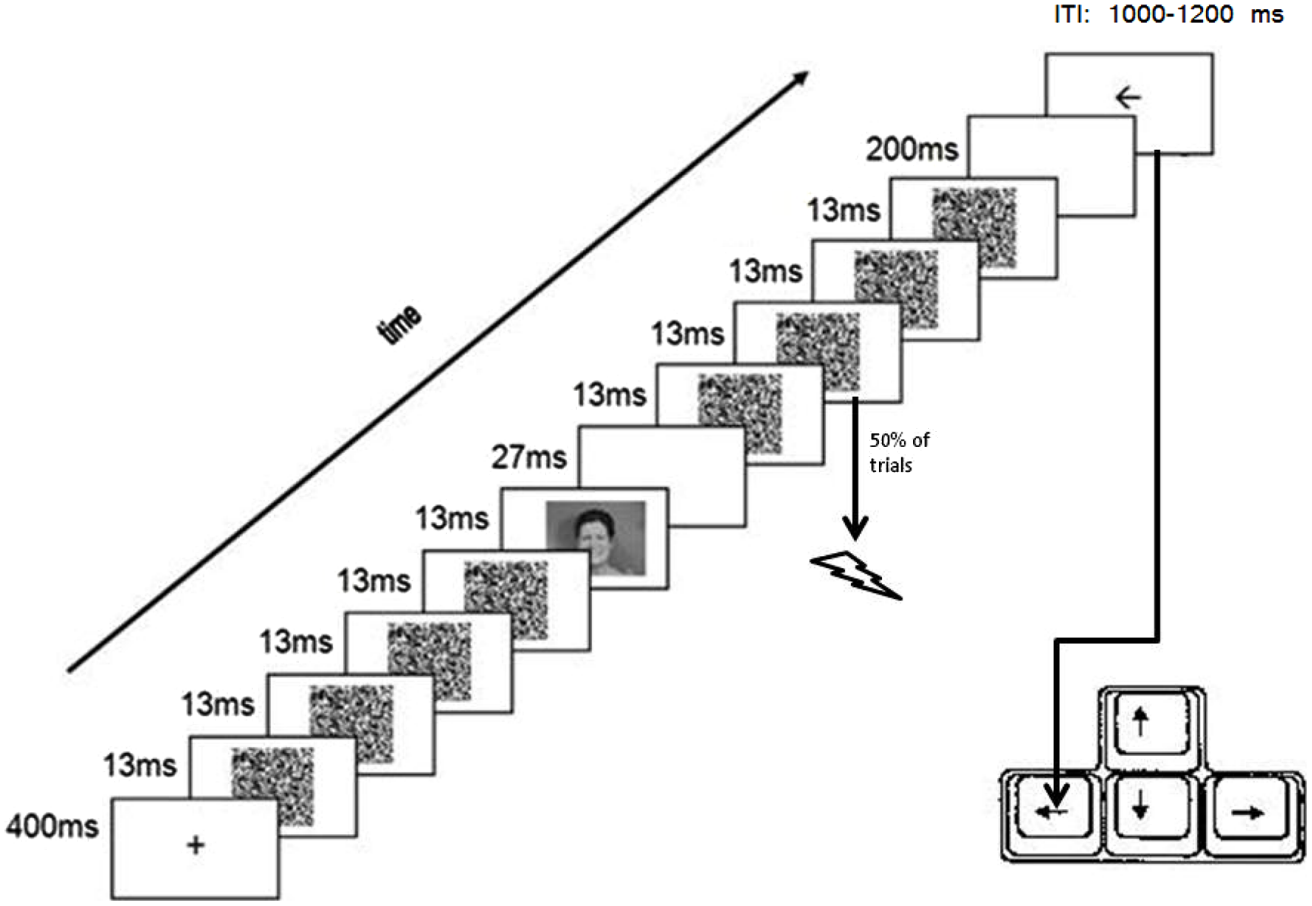
**Configuration of a typical trial.** Response was given using the dominant hand and electrical stimuli were delivered to the non-dominant hand.

**Figure 1** presents a typical trial configuration which was based on previous masked priming studies (e.g., Dell’Acqua and Grainger, 1999; Van den Bussche et al., 2009). Throughout the task, all stimuli appeared at central fixation on a gray background (RGB: 150, 150, 150). All stimulus presentations were synchronized with the vertical refresh cycle of the screen (13.3 ms). Each trial started with a small (1 mm*1 mm) black fixation cross for 400 ms. Then, a masked photograph of a facial expression (i.e., the *prime*) was presented (cf. Delord, 1998; Van den Bussche et al., 2009). More specifically, the fixation cross was first replaced by a series of four different masks (random black-and-white dot patterns; width = 9 cm, Height = 6.5 cm), each presented for 13.3 ms. Immediately after the offset of the fourth mask, a facial expression was presented for 13.3 ms, after which a blank was presented for 27 ms. Then, a series of four masks was presented again. At the onset of the second mask in this series the electrocutaneous stimulus was delivered on half of the trials (randomly determined); during the other half of the trials no electrocutaneous stimulus was delivered. Immediately after the offset of the last mask, a blank was presented for 200 ms. Finally, the *target*, a black arrow, was presented (width = 8 cm, Height = 5.5 cm). On half of the trials (for both trials with and without electrocutaneous stimulation) the arrow pointed to the right; on the other half of the trials the arrow pointed to the left. Participants were instructed to classify the arrow as fast as possible by pressing the corresponding arrow keys on the bottom right of an AZERTY keyboard with their dominant hand, while avoiding mistakes. The arrow was presented until one of the response keys was pressed or for a maximum of 3000 ms. The arrow was followed by an inter-trial interval that randomly varied between 1000 and 1200 ms (could be either 1000, 1100, or 1200 ms) and during which the screen was blank.

Participants were not informed about the presence or the type of the facial expressions. The three facial expression types were presented in three separate blocks. Each block contained 48 trials with each of the four faces presented 12 times in each block (six trials with electrocutaneous stimuli and six times without). Block order was counterbalanced between participants. After each block, participants were asked to rate the average intensity, unpleasantness, and painfulness of the electrocutaneous stimulation experienced during the previous block on three separate 100 mm visual analog scales with the end points labeled ‘0 = not intense/unpleasant/painful at all’ and ‘10 = extremely intense/unpleasant/painful.’ Breaks between blocks were self-paced.

#### Prime Awareness Check

To determine participants’ objective awareness of the sub-optimally presented facial expressions (i.e., the primes), a forced-choice prime awareness task was administered after the priming task (Van den Bussche et al., 2009). In this task a fixation cross appeared on the screen (400 ms) and replaced by four consecutive masks (13.3 ms each). Then a facial expression was presented for 27 ms and replaced by a blank screen (13.3 ms) which was followed by a series of four masks (13.3 ms each). After the last mask three Dutch words appeared on the screen (Font 28 Arial, in Black, First letter capitalized, 5 cm below the fixation cross and interspaced by 5 cm). These words were “painful” (“pijnlijk”), “happy” (“blij”), and “neutral” (“neutraal”). Participants were explicitly informed that a sub-optimal facial expression was presented on each trial and they were asked to classify that by mouse-clicking the corresponding word. Words were presented until a response was given and after each trial the position of the cursor was returned to the center of the screen. Participants were instructed to guess if they could not see the facial expression. The three facial expression types were presented in a randomized manner (each expression was presented four times, so the task had total of 48 trials). If participants were unaware of the primes, this was indicated by performance at chance level (i.e., 33%) on this prime awareness task.

### Apparatus

Electrocutaneous stimulus delivery, task presentations, and logging of button presses were controlled by a Dell Optiplex 755 computer (OS: windows XP; 2 GB RAM; Intel Core2 Duo processor at 2.33 GHz; ATI Radeon 2400 graphics card with 256 MB of video RAM), running Affect 4.0 software (Spruyt et al., 2010) and connected to a 19” CRT DELL monitor (75 Hz vertical refresh rate; refresh duration: 13.3 ms/frame), an AZERTY keyboard, a mouse, and a constant current stimulator (see above).

### Procedure

All participants were tested individually in a dimly lit testing room. They were video-monitored and could communicate via an intercom with the experimenter who was located in a separate room. Upon arrival at the testing room, they received an information sheet describing the experimental procedure. More specifically, it was explained that the study focused on the factors involved in the perception of pain. Participants were informed that they would perform a simple categorization task while receiving painful electrocutaneous stimuli. Then they signed the informed consent and completed demographic questions and a battery of Dutch questionnaires including the PCS and the FPQ. After questionnaire completion, electrodes were attached and painful electrocutaneous stimulus intensity was individually set. Then participants performed the priming task followed by the objective prime awareness check. Finally, the electrodes were detached and participants were debriefed and informed about the purpose of the experiment.

## Results

### Participant Characteristics

**Table 1** presents an overview of participants’ scores on the questionnaires. The PCS and FPQ ratings of the present sample are comparable to PCS and FPQ ratings of similar samples in previously published studies (Van Damme et al., 2000; Roelofs et al., 2005; Engelen et al., 2006).

**Table 1:** Participants’ mean scores on the questionnaires (*N* = 22).

Questionnaires	Total score/Subscale	Mean	Median	SD	Minimum	Maximum
Pain Catastrophizing Scale (PCS)	Total	14.64	13.50	10.03	0	30
	Rumination	6.86	8.00	4.70	0	13
	Magnification	3.18	2.00	2.48	0	9
	Helplessness	4.59	4.00	4.01	0	12
Fear of Pain Questionnaire (FPQ)	Total	68.77	65.50	13.47	46	96
	Severe pain	32.77	33.00	5.99	20	42
	Minor Pain	15.36	15.50	4.52	10	29
	Medical Pain	20.64	18.50	5.83	13	36

### Priming Task Performance

This section focuses on RT analyses^1^ Incorrect responses (*M* = 2.5%, SD = 2.1) and responses slower than 1000 ms (less than 1% of the trials) were removed prior to RT analyses. In addition, we noticed that due to a software failure, during 20.8% of trials the presentation time for at least one stimulus (a mask, the prime, or the blank presented after the prime) was zero instead of 13 ms, so these trials were removed from the analyses as well. After removing these trials, there were at least 14 (*M* = 18.8, SD = 0.7, range: 14–23 trials) trials for each subject during each block and each condition which was sufficient for the purpose of analyses. The reported analyses were performed on mean RTs.

Mean RTs were subjected to a repeated-measures ANOVA with electrocutaneous stimulation (two levels: aversive electrocutaneous stimulation vs. no electrocutaneous stimulation) and facial expression type (three levels: painful vs. happy vs. neutral) as within subjects factors. Mean RTs (SD) as a function of electrocutaneous stimulation and facial expression type are presented in **Table 2**.

**Table 2:** Reaction times in function of prime type (happy, neutral, or painful) and electrocutaneous stimulus presence (Yes or No).

		Reaction times				
Electrocutaneous stimulus present	Prime type	Mean	Median	SD	Minimum	Maximum
Yes	Happy	335.39	328.17	28.82	291.81	404.15
	Neutral	338.99	337.55	30.60	275.43	409.47
	Painful	329.78	325.95	29.54	273.43	401.52
No	Happy	343.54	346.80	26.32	305.05	404.11
	Neutral	340.25	341.15	25.73	282.30	394.94
	Painful	351.15	346.66	39.04	277.33	443.05

There was a significant main effect of electrocutaneous stimulation [*F*(1,21) = 15.90, *p* = 0.001, $\eta_{p}^{2}$ = 0.43] with faster RTs to targets preceded by aversive electrocutaneous stimulation (*M* = 334.7 ms, SD = 29.4) than to targets preceded by no electrocutaneous stimulation (*M* = 345.0 ms, SD = 30.8). There was no main effect of facial expression type [*F*(2,42) = 0.20, *p* = 0.90, $\eta_{p}^{2}$ = 0.001]. However, a significant interaction between electrocutaneous stimulation and facial expression type emerged [*F*(2,42) = 4.57, *p* = 0.02, $\eta_{p}^{2}$ = 0.18].

.

**FIGURE 2:**
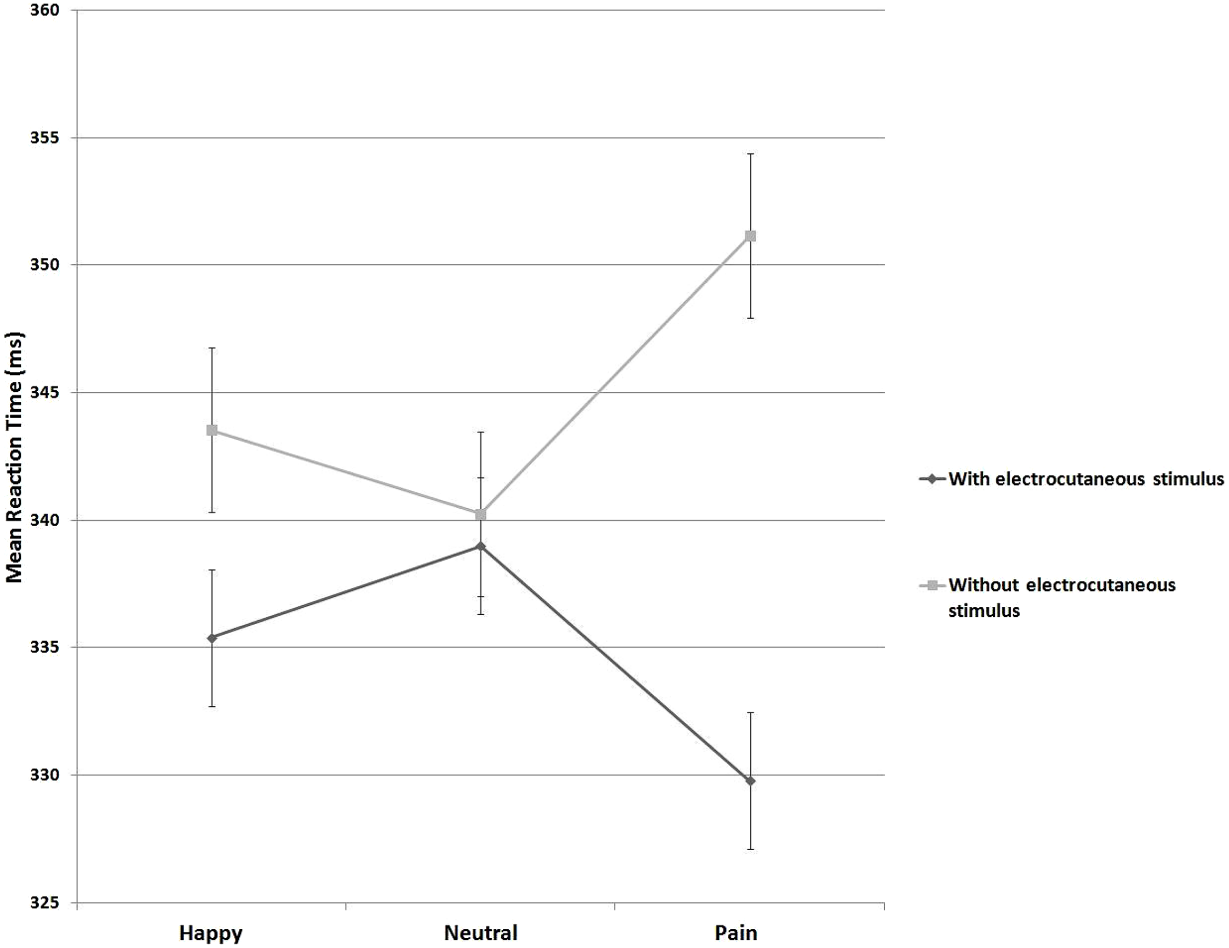
**Mean reaction times (RTs) on trials with and without electrocutaneous stimulus in three blocks with different primes (Happy, Neutral, Painful)**.

In order to address this significant interaction, an index of response facilitation was computed by subtracting mean RT to targets preceded by aversive electrocutaneous stimulation from RTs to targets preceded by no electrocutaneous stimulation. A *post hoc t*-test, comparing this index against zero (i.e., no response facilitation) indicated response facilitation for targets preceded by electrocutaneous stimulation following painful expressions [*M* = 21.4, SD = 24.2, *t*(21) = 4.12, *p* < 0.001, *Cohen’s d* = 0.98]. However, following happy [*M* = 8.1, SD = 20.5, *t*(21) = 1.81, *p* = 0.08, *Cohen’s d* = 0.40] and neutral expressions [*M* = 1.2, SD = 20.8, *t*(21) = 0.28, *p* = 0.78, *Cohen’s d* = 0.06] no significant response facilitation emerged (**Figure 2**).

The observed facilitation of responses in trials with painful expressions was significantly different from trials with neutral expression [*t*(21) = 2.76, *p* = 0.01, *Cohen’s d* = 0.59]. There was no such a difference between trials with happy expressions and neutral expressions [*t*(21) = 1.31, *p* = 0.21, *Cohen’s d* = 0.28], nor between painful expressions and happy expressions [*t*(21) = 1.75, *p* = 0.09, *Cohen’s d* = 0.37].

Inclusion of PCS or FPQ as centered covariate into the ANOVA described above did not change the reported pattern of results and did not reveal any new main effect or interaction.

### Pain Rating

**Table 3** provides an overview of mean (SD) ratings of painfulness, intensity and unpleasantness separately for each facial expression type.

**Table 3:** Participants mean ratings (*M* ± SD) of electrocutaneous stimulus after each block of the priming task (*N* = 22).

	Pain rating	Electrocutaneous stimulus intensity	Electrocutaneous stimulus unpleasantness
**Prime type**			
Painful	5.82 ± 1.94	5.68 ± 2.01	6.18 ± 1.82
Happy	5.18 ± 2.36	5.23 ± 2.31	5.72 ± 2.03
Neutral	5.55 ± 1.87	5.45 ± 2.06	6.00 ± 1.83

Ratings of painfulness, intensity, and unpleasantness were subjected to three separate repeated measures ANOVAs with facial expression type (three levels: painful, happy, neutral) as within-subjects factor.

For painfulness ratings, the main effect of facial expression type shows a trend toward significance [*F*(2,42) = 2.81, *p* = 0.07, $\eta_{p}^{2}$ = 0.12). Mean painfulness ratings were higher following painful expressions (*M* = 5.81, SD = 1.9) than following happy expressions (*M* = 5.18, SD = 2.4) [*t*(21) = 2.08, *p* = 0.05, *Cohen’s d* = 0.44]. There were no significant differences between painfulness ratings following neutral expressions (*M* = 5.55, SD = 1.9) and either happy [*t*(21) = 1.40, *p* = 0.18, *Cohen’s d* = 0.30] or painful expressions [*t*(21) = 1.14, *p* = 0.27, *Cohen’s d* = 0.24].

For intensity ratings, the effect of facial expression type did not reach statistical significance [although it showed a trend: *F*(2,42) = 2.69, *p* = 0.09, $\eta_{p}^{2}$ = 0.11]. Mean intensity ratings were higher following painful expressions (*M* = 5.68, SD = 2.0) than following happy expressions (*M* = 5.23, SD = 2.3) [*t*(21) = 2.08, *p* = 0.06, *Cohen’s d* = 0.43], though this comparison also did not reach significance. There were no differences between intensity ratings following neutral expressions (*M* = 5.45, SD = 2.1) and either happy [*t*(21) = 1.31, *p* = 0.2, *Cohen’s d* = 0.28] or painful expressions [*t*(21) = 1.23, *p* = 0.2, *Cohen’s d* = 0.26].

For unpleasantness ratings, there was no significant main effect of facial expression type [*F*(2,42) = 1.66, *p* = 0.26, $\eta_{p}^{2}$ = 0.13].

### Prime Awareness Check

Overall prime awareness was 37% which was not significantly higher than chance level (i.e., 33%), [*t*(21) = 1.54, *p* = 0.14, *Cohen’s d* = 0.36], suggesting that on average participants were not aware of whether a painful, happy, or neutral expression was presented and that facial expressions were presented sub-optimally.

## Discussion

In the current study, we aimed to investigate the effect of sub-optimally presented pictures of painful, happy and neutral facial expressions on action readiness and ratings of painfulness, intensity, and unpleasantness of the electrocutaneous stimulation.

The results can be readily summarized. First, responses to non-pain-related targets were faster following electrocutaneous stimulation than when no stimulation was delivered, indicating enhanced readiness for action. Second, this response facilitation was greater when the electrocutaneous stimulus was preceded by a sub-optimally presented painful expression compared to happy or neutral expressions. Third, painfulness ratings were higher following painful expressions than following happy expressions.

Faster responses to targets preceded by aversive electrocutaneous stimulation than to targets not preceded by stimulation were taken to reflect improved action readiness following aversive tactile stimulation (cf. van Loon et al., 2010). This is in line with findings of a previous study which provided evidence in support of a hypothesis on a higher cortico-spinal excitability when observing unpleasant compared to pleasant or neutral stimuli, and no difference in the excitability when observing neutral compared to pleasant stimuli (van Loon et al., 2010). To our knowledge, our study is the first study investigating the effect of aversive electrocutaneous stimulation in combination with sub-optimal processing of painful and non-painful facial expressions on the observer’s readiness for taking an action in an unrelated behavioral task. The observation of enhanced action readiness following aversive tactile stimulation is in line with the cognitive motivational priming hypothesis which predicts that when we encounter threat, a defensive system automatically increases our readiness to reduce the consequences of such an encounter (Lang, 1995). In a similar vein, it has been suggested that activation of low-level self-defensive mechanisms by perceived threat from electrocutaneous stimulation can activate brain areas responsible for preparation of an action (e.g., premotor cortex) through a projection from the brain areas involved in the affective evaluation of perceived stimuli (Buchel et al., 1998) which might lead to faster responses.

The present data revealed enhanced action readiness following the sub-optimal presentation of painful expressions. This finding might have implications for research on human empathy, suggesting that observation of pain in the facial expression of another person results in increased readiness in the observer for taking action. The facilitation in the responses is corroborated by the finding that empathic responses to painful facial expressions are primarily influenced by the threat value of pain, and that perceived threat encourages faster reactions (Yamada and Decety, 2009). Although previous studies have demonstrated the enhancing impact of clearly visible optimally presented painful facial expressions on action readiness (Vachon-Presseau et al., 2011, 2012; Mailhot et al., 2012; Khatibi et al., 2014), the present study is the first demonstration of the impact of sub-optimally presented painful facial expressions on action readiness. We used a masking paradigm to prevent the expressions from being fully consciously processed by the observer. Previous researches have shown that masked primes can be processed up to a semantic level (Van den Bussche and Reynvoet, 2007; Van den Bussche et al., 2009). In addition, it has been shown that processing of emotion in expressions is a rapid and automatic process which starts at the early stages of processing (Batty and Taylor, 2003; Ibanez et al., 2011). These authors also suggested that differentiation of different emotions in the expressions starts at those early stages of processing and is not limited to the processing at the strategic level.

Complementary to the literature and comparing findings of this study with previous studies which used emotional priming by presentation of emotional facial expressions at optimal processing condition may suggest that conscious processing of emotional (here painful and happy) facial expressions is not necessary for the semantic processing of those expressions. Accordingly, we can assume that the presentation of painful facial expressions under a condition of restricted awareness in our study did not interfere with the processing of the threatening value of these expressions by observers, although the subjects were not able to consciously report or identify them. In line with the literature our observation suggests that the processing of (threat in) painful facial expressions does not need to be performed at a fully conscious level to influence the observer’s subsequent actions and that even sub-optimally presented facial stimuli can improve the readiness for an action in the observer.

It should be noted that RTs on trials with painful expressions and electrocutaneous stimulation were faster than on trials with painful expressions but without electrocutaneous stimulation (this difference for the other two types of expressions did not reach significance). The observed interaction between the effect of processing of pain in others and processing of an electrocutaneous painful stimulus can be further explained in the light of theories on the empathy. These theories hypothesize that one of the functions of empathy in human is toward the preparation of the person for coping with potential demands of the situation (Preston and de Waal, 2002). It has been shown that the processing of visual cues which signal the presence of an impending threat can activate defensive mechanisms which prime motor responses (Mulckhuyse and Crombez, 2014). Previous studies also suggested that observation of pain in facial expressions of others can be seen as a signal for an impending threat (Williams, 2002). In addition, a congruent presentation of a visual cue, which signals threat, with a somatosensory cue (electrocutaneous stimulation) improves subjects’ readiness for taking an action (Mulckhuyse and Crombez, 2014). One possible but still speculative explanation about the observed interaction is that painful facial expressions increased readiness for taking an action and when it is paired with aversive electrocutaneous stimulation resulted in increased excitability and thus faster responses through the congruency between visual cue and sensory cue (Mulckhuyse and Crombez, 2014). On the other hand, the absence of aversive electrocutaneous stimulation after painful facial expressions makes this condition an incongruent condition. This means that the readiness state activated by observation of pain in others needs to be suppressed because anticipation for electrocutaneous stimulation following the processing of the expression was not validated. This would inhibit the activated excitation to bring the response system back to its pre-activation level, resulting in slower responses.

Our results showed that participants’ painfulness ratings were slightly higher following painful expressions than following happy expressions. This finding is in line with previous studies demonstrating that pain perception can be influenced by observation of pain in others (Vachon-Presseau et al., 2011; Mailhot et al., 2012; Reicherts et al., 2013; Khatibi et al., 2014). It is suggested that activation of the brain during the observation of pain in others is similar to the brain’s response to the first hand experience of pain (Botvinick et al., 2005; Saarela et al., 2007). It has been suggested that activation in brain areas in response to the observation of pain in others may facilitate processing of pain in the observer which can result in higher pain perception in the observer (Vachon-Presseau et al., 2011; Mailhot et al., 2012). However, this explanation is based on findings of behavioral and neuropsychological studies and none of previous studies directly tested this hypothesis. Future brain imaging studies may help us to test this in a more direct manner.

Some study limitations and suggestions for future research should be noted. First, our participants rated the electrocutaneous stimuli retrospectively following each block of trials. Retrospective ratings are more prone to be influenced by memory bias than online ratings upon stimulation (Redelmeier and Kahneman, 1996). Second, our sample mainly composed of female participants. A larger and more (gender) balanced sample would be helpful to explore the generalizability of our results. Third, although problems related to the physical and psychological health (such as chronic pain problems or history of mental disorders) were considered as exclusion criteria, we did not include specific measures to test them in our subjects. Future studies may benefit from these measures to have a more homogenous sample. Fourth, in the current experiment we only included emotional expressions related to pain and not to other negatively valenced stimuli. Although some previous studies have shown that observation of other negative emotions (such as sad faces) can increase pain perception (Bayet et al., 2014), but it is not investigated whether they can influence action readiness or not. This is something that needs to be investigated in future research to test the specificity of the effect we observed in the current study. Finally, action readiness was assessed for simple classification responses. This task does not represent an approach or avoidance oriented action. The literature of research on the empathy has widely discussed the importance of observation of emotion in others and selection of approach oriented action (altruistic behavior) or avoidance oriented action (defensive behavior; Preston and de Waal, 2002). Activation of any of these two mechanisms is dependent upon a number of other factors (e.g., the relationship between the observed person and the observer, contextual factor, and etc). Future studies should use more complex tasks to investigate the effect of the observation of painful facial expressions on the performance in more cognitive demanding situations and to differentiate its effect on the activation of approach or avoidance oriented actions.

## Conclusion

Sub-optimal presentation of painful facial expressions facilitated observers’ responses on a non-pain-related behavioral task when these expressions were followed by electrocutaneous stimulation. Furthermore, the painful expressions increased participants’ perception of painfulness of the electrocutaneous stimulation. This is in accordance with literature on the vicarious facilitation of responses and shows that this facilitation can also occur under sub-optimal observation conditions.

## Conflict of Interest Statement

The authors declare that the research was conducted in the absence of any commercial or financial relationships that could be construed as a potential conflict of interest.

## Footnotes

1 There was not enough variability in the error rates to allow for parametric analyses (see Supplementary Table S1 in supplementary materials).
