# Supplementary material for: Sub-optimal presentation of painful facial expressions enhances readiness for action and pain perception following electrocutaneous stimulation
Source: Front Psychol. 2015 Jul 7;6:913. doi: 10.3389/fpsyg.2015.00913 (PMC4493322; doi:10.3389/fpsyg.2015.00913)
Supplement: Supplementary file 1 [file Table_1.DOCX]

| **Table S1**  *Percentage of errors in Function of Prime Type (Happy, Neutral or Painful) and Electrocutaneous stimulus Presence (Yes or No).* | | | | | | | | |
| --- | --- | --- | --- | --- | --- | --- | --- | --- |
|  |  | *Errors* | | | | | | |
| Electrocutaneous stimulus present | Prime type | *Mean%* | *SD%* | | *Min%* | | *Max%* |  |
|  |  |  |  |  |  |  |  |  |
| Yes | Happy | 4.04 | | 4.4 | | 0 | 12.5 | |
|  | Neutral | 2.65 | | 3.6 | | 0 | 14.3 | |
|  | Painful | 1.81 | | 3.3 | | 0 | 9.9 | |
| No | Happy | 2.07 | | 2.7 | | 0 | 8.7 | |
|  | Neutral | 2.14 | | 3.2 | | 0 | 10.5 | |
|  | Painful | 2.18 | | 3.6 | | 0 | 12.5 | |
